# Development of Effective Antibacterial Treatment: Lessons from the Past and Novel Approaches

**DOI:** 10.3390/antibiotics10030230

**Published:** 2021-02-25

**Authors:** Mariagrazia Di Luca, Tiziano Marzo

**Affiliations:** 1 Department of Biology, University of Pisa, Via San Zeno 35-39, 56100 Pisa, Italy; 2 Department of Pharmacy, University of Pisa, Via Bonanno Pisano 6, 56126 Pisa, Italy

In the last three decades, the appearance and rapid diffusion of antibiotic-resistant bacterial strains have been observed. Contextually, the discovery rate of novel antibacterial agents has declined steeply. Hence, the overuse/misuse of antimicrobials along with the lack of development of new effective molecules has led to the so-called “antibiotic resistance crisis”. Within this frame, active substances and alternative strategies are urgently needed to fight this global threat to public health. The development of novel active molecules is being intensively pursued, but this approach is time-consuming and very expensive. Thus, testing novel associations of already approved drugs and drug repositioning may represent valuable tools to addressing the problem. Moreover, rediscovering older antimicrobial therapies, such as treatment based on bacteriophages which are active against multidrug-resistant bacteria, could be a promising approach for the therapy of difficult-to-treat infections. The combination of antibiotics together with bacteriophages or other molecules has been also successfully exploited. Finally, several promising targeted and responsive drug delivery platforms aiming to enhance the antibacterial properties have been recently tested. This Special Issue consists of five research articles and three reviews on the above topics that are crucial for the field of development of antibacterial agents.

This collection of manuscripts includes papers published in the Special Issue of *Antibiotics* “Development of Effective Antibacterial Treatment: Lessons from the Past and Novel Approaches”. First, we present two systematic reviews dealing with the application of phage therapy for the treatment of both bone and joint infections and for superficial bacterial infections [1,2]. Phage therapy is a promising antibacterial strategy suitable for infections featuring a biofilm component. Through a search based on three electronic databases, Jones et al. summarize the clinical results in terms of the effectiveness of phage therapies in two eligible patient cohorts. In the first cohort including patients with bone and joint infections, the use of appropriately purified phages emerges as a suitable safe and highly efficacious treatment option for complex and intractable infections. The outcome from the second review clearly suggests as purified phage can be highly effective in the treatment of superficial bacterial infections, and that by using various routes of administration, it is safe and tolerable for patients. The third review by Marasco and coworkers is a comprehensive overview of the role of new therapeutic agents and antimicrobial peptides against tuberculosis. In this paper, authors focus their attention on the most recent findings in the field of metal complex–peptide conjugates and their delivery systems with potential pharmaceutical applications as novel antibiotics in *Mycobacterium tuberculosis* infections [3].

The research article by the group of Philipp Uhl concerns peptides used as antimicrobial agents. Specifically, the synthetic peptide Pep19-4LF (GKKYRRFRWKFKGKLFLFG) is optimized through covalent binding to saturated fatty acids of different chain lengths. Interestingly, the length of the fatty acid is directly correlated to the antibacterial potency without toxic effects. Preliminary in vivo experiments in animal models demonstrated pharmacokinetics appropriate for application as a drug, confirming that the hydrophobic chain of the peptide can be replaced by a single fatty acid, simplifying its design while retaining antimicrobial activity [4].

In the paper by Yang et al., the authors focus their attention on methicillin-resistant *Staphylococcus aureus* (MRSA). Indeed, it is often necessary to treat this kind of infection using a combination of different antibiotics to overcome antibiotic resistance while avoiding the development of greater antibiotic resistance. In this frame, a strategy relying on the use of free fatty acids and surfactants in combination with oxacillin is investigated. The optimal ratio of these three components and the synergistic effects of possible combinations were investigated. Results reveal that by combining oxacillin with palmitic acid and a surfactant (span85), the same antibacterial potency is achieved using a lower concentration of oxacillin and reducing the possibility of acquiring drug resistance [5].

The treatment for *Staphylococcus aureus* infections, specifically implant-associated bone infections, is the topic of the paper by Trampuz and coworkers. They propose the use of staphylococcal bacteriophage Sb-1 combined with five different antibiotics to exploit synergistic activity against biofilm infections. Five antibiotics, i.e., doxycycline, levofloxacin, clindamycin, linezolid, and rifampin, alone or in combination with Sb-1, are tested against six strains. Different effects are reported, including some very promising synergistic effects in all the strains, namely in the case of phage/doxycycline and phage/linezolid combinations. Overall, these results are relevant for an alternative, combined, and prolonged suppressive antimicrobial treatment strategies [6]. The paper by Howe et al. deals with the use of Cu(II)-, Mn(II)-, and Ag(I)-based complexes for the treatment of chronic *Pseudomonas aeruginosa* infections in cystic fibrosis (CF) patients. Particularly critical is the capability of the bacteria to form biofilms that, in turn, determines decreased susceptibility to most antibiotic treatments. Complexes are assayed using clinical isolates of *P. aeruginosa* from Irish CF patients in comparison with two reference laboratory strains (ATCC 27853 and PAO1). The complexes show comparable or superior activity to gentamicin in the CF strains compared with their activities in the laboratory strains, with respect to both biofilm formation and established biofilms. Synergistic activity of the metal-based compounds with gentamicin is also reported [7]. Cavalli et al. developed an interesting strategy to treat multidrug-resistant (MDR) Gram-negative bacteria (GNB) that relies on the delivery of colistin encapsulated in multifunctional chitosan-coated human albumin nanoparticles (Col/haNPs). After an extensive characterization, the Col/haNPs were assayed in vitro for their antibacterial activity against MDR GNB (*Acinetobacter baumannii* and *Klebsiella pneumoniae*). Col/haNPs feature high tolerability accompanied by a high antibacterial effect compared to free colistin. Importantly, the effects are reported against Col-resistant strains. Moreover, the colistin-loaded nanoparticles exhibit inhibition of biofilm formation. Altogether, these findings indicate that Col/haNPs are a promising colistin nanocarrier with potent antimicrobial activity on MDR GNB [8].

*The Guest Editors are greatly thankful to all the authors and reviewers. We are very grateful to the Antibiotics Editorial Office for giving us the opportunity to organize and support this Special Issue.*

## References

[1] Clarke A.L., De Soir S., Jones J.D. (2020). The Safety and Efficacy of Phage Therapy for Bone and Joint Infections: A Systematic Review. Antibiotics.

[2] Steele A., Stacey H.J., de Soir S., Jones J.D. (2020). The Safety and Efficacy of Phage Therapy for Superficial Bacterial Infections: A Systematic Review. Antibiotics.

[3] Di Natale C., De Benedictis I., De Benedictis A., Marasco D. (2020). Metal–Peptide Complexes as Promising Antibiotics to Fight Emerging Drug Resistance: New Perspectives in Tuberculosis. Antibiotics.

[4] Storck P., Umstätter F., Wohlfart S., Domhan C., Kleist C., Werner J., Brandenburg K., Zimmermann S., Haberkorn U., Mier W. (2020). Fatty Acid Conjugation Leads to Length-Dependent Antimicrobial Activity of a Synthetic Antibacterial Peptide (Pep19-4LF). Antibiotics.

[5] Song H.-S., Choi T.-R., Bhatia S.K., Lee S.M., Park S.L., Lee H.S., Kim Y.-G., Kim J.-S., Kim W., Yang Y.-H. (2020). Combination Therapy Using Low-Concentration Oxacillin with Palmitic Acid and Span85 to Control Clinical Methicillin-Resistant *Staphylococcus aureus*. Antibiotics.

[6] Wang L., Tkhilaishvili T., Trampuz A. (2020). Adjunctive Use of Phage Sb-1 in Antibiotics Enhances Inhibitory Biofilm Growth Activity versus Rifampin-Resistant Staphylococcus aureus Strains. Antibiotics.

[7] O’Shaughnessy M., McCarron P., Viganor L., McCann M., Devereux M., Howe O. (2020). The Antibacterial and Anti-Biofilm Activity of Metal Complexes Incorporating 3,6,9-Trioxaundecanedioate and 1,10-Phenanthroline Ligands in Clinical Isolates of Pseudomonas aeruginosa from Irish Cystic Fibrosis Patients. Antibiotics.

[8] Scutera S., Argenziano M., Sparti R., Bessone F., Bianco G., Bastiancich C., Castagnoli C., Stella M., Musso T., Cavalli R. (2021). Enhanced Antimicrobial and Antibiofilm Effect of New Colistin-Loaded Human Albumin Nanoparticles. Antibiotics.

